# Blunt Thoracic Aortic Injury Presenting as Hemodynamically Stable: A Case Report

**DOI:** 10.5811/cpcem.53232

**Published:** 2026-07-20

**Authors:** Joseph Knudsen, Stephen Lucas, James Mangano

**Affiliations:** State University of New York Upstate Medical University, Department of Emergency Medicine, Syracuse, New York

**Keywords:** blunt thoracic aortic injury, motor vehicle collision, hemodynamic stability, case report

## Abstract

**Introduction:**

Blunt thoracic aortic injury is a rare but potentially fatal consequence of motor vehicle collisions. While commonly associated with hemodynamic instability, some cases present with normal vital signs, delaying diagnosis and treatment. We present the case of a 42-year-old unrestrained backseat passenger who sustained a blunt thoracic aortic injury following a motor vehicle collision and underwent emergent endovascular repair. This case emphasizes the critical role of early imaging and multidisciplinary coordination in the management of high-risk trauma patients.

**Case Report:**

A 42-year-old female presented to the emergency department (ED) following a high-risk motor vehicle collision. She was an unbelted passenger in a vehicle traveling at approximately 35 miles per hour that collided with a telephone pole. The patient was unconscious at the scene but regained consciousness en route. Upon arrival at the ED she was alert, oriented, and hemodynamically stable. Her vital signs were as follows: blood pressure, 107/58 millimeters of mercury; heart rate, 62 beats per minute; respiratory rate, 18 breaths per minute; and oxygen saturation, 100% on room air. Physical examination revealed anterior chest wall tenderness but no signs of respiratory distress. Imaging revealed a high-grade blunt thoracic aortic injury with a left-sided hemothorax. The patient underwent emergent thoracic endovascular aortic repair and remained hemodynamically stable throughout hospitalization.

**Conclusion:**

Blunt thoracic aortic injury can present with hemodynamic stability despite its life-threatening nature. This case highlights the necessity of early imaging and multidisciplinary coordination in optimizing outcomes for high-risk trauma patients.

## INTRODUCTION

Blunt thoracic aortic injury is the second most common cause of death in blunt trauma.^1^,^2^ It is frequently associated with motor vehicle collisions, as this is a common mechanism of injury for blunt thoracic aortic injury.^3^ The classic presentation involves rapid cardiovascular decompensation due to massive hemorrhage. However, cases of hemodynamic stability despite significant injury can occur, which may delay diagnosis and treatment.^4^ This report describes a case of a patient with a high-grade blunt thoracic aortic injury with normal vital signs, highlighting the importance of early imaging and prompt surgical intervention.

## CASE REPORT

A 42-year-old female presented to the emergency department (ED) at approximately 8:30 am following a high-risk motor vehicle collision. She was an unrestrained backseat passenger in a vehicle that struck a telephone pole at approximately 35 miles per hour. Emergency medical services (EMS) reported that the patient was initially unconscious but regained consciousness en route. Upon arrival, she was alert and oriented, and not in acute distress. Her vital signs were as follows: temperature, 36.1 °C; blood pressure, 107/58 millimeters of mercury (mm Hg); heart rate, 62 beats per minute; respiratory rate, 18 breaths per minute, and oxygen saturation 100% on room air. Her body mass index was 22.14 kilograms per square meter (kg/m^2^) (reference range: 18.5–24.9 m^2^), and her past medical history included chronic neck and back pain, fibromyalgia, opioid abuse, and anxiety and depression.

Physical examination revealed diffuse anterior chest wall tenderness, particularly over the sternum. Nasal bridge tenderness with overlying ecchymosis and dried blood in the bilateral nares was noted, without septal hematoma. There was no midline cervical, thoracic, or lumbar spine tenderness, no respiratory distress, and clear bilateral breath sounds.

Initial laboratory evaluation obtained at arrival demonstrated leukocytosis (white blood cell count 22.8 × 10^9^ per liter (10^9^/L)(4.0–11.0 ×10^9^/L)) and anemia (hemoglobin 10.3 grams per deciliter [g/dL] [12.0–16.0 g/dL]; hematocrit 29.6% [36–46%]), without evidence of coagulopathy (international normalized ratio 1.22 [0.8–1.2]) or renal dysfunction (creatinine 0.88 mg/dL [0.6–1.3 mg/dL]). Mild transaminase elevations were present (aspartate aminotransferase 194 units/L [10–40 U/L]); alanine aminotransferase 137 U/L [7–56 U/L].

**Image 1:**
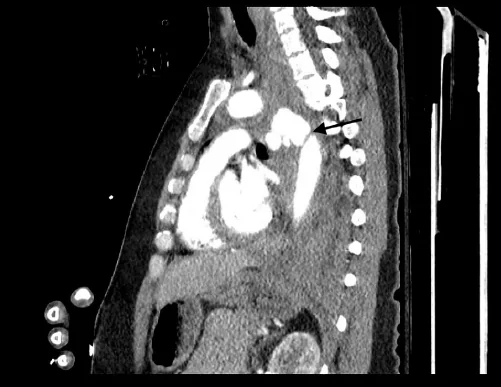
Computed tomography of the chest with contrast in sagittal view demonstrating the full extent of the aortic transection (arrow). Findings are consistent with a grade four blunt thoracic aortic injury. The transection extends into the proximal portion of the descending aorta.

**Image 2:**
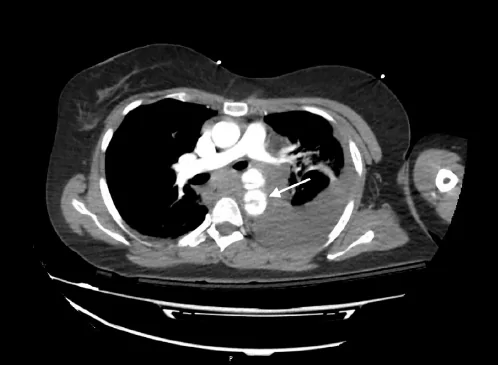
Computed tomography of the chest with contrast in axial view demonstrating transection of the aortic wall (arrow).

**Image 3:**
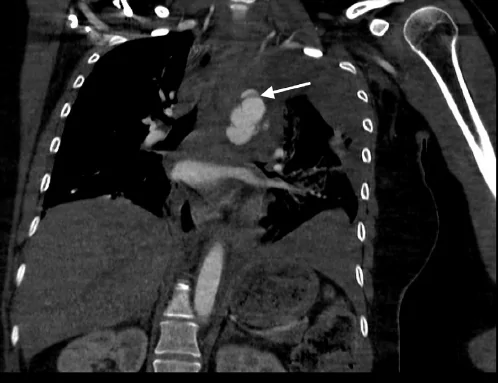
Computed tomography of the chest with contrast in coronal view demonstrating disruption of the descending thoracic aorta (arrow), consistent with blunt thoracic aortic injury.

Initial chest radiograph revealed diffuse opacities in the left lung with complete opacification of the inferior segments, concerning for pulmonary contusion and hemothorax. Given the high-energy mechanism of injury and concern for thoracic trauma, a computed tomography (CT) chest with contrast was obtained at 10:00. Computed tomography revealed a grade four blunt thoracic aortic injury with a significant left-sided hemothorax (Images 1–3). The severity of the aortic injury and associated hemothorax prompted immediate consultation with trauma and vascular surgery teams. Due to stable hemodynamics and relative bradycardia, anti-impulse therapy was considered but not initiated in the ED, with priority given to rapid operative management. Analgesia was initiated with fentanyl (50 micrograms intravenous [IV] at 9:15 am and 10:10 am), and ondansetron 4 mg IV was given at 10:03 am for nausea prophylaxis.

Additional injuries included nasal bone fractures and grade 1 liver and splenic lacerations, which were managed conservatively. The patient underwent thoracic endovascular aortic repair at 11:24 am with placement of an endograft covering the transected aortic segment as well as partial coverage of the left subclavian artery to ensure adequate seal zone. A left-sided surgical chest tube was placed for management of the hemothorax, with approximately 700 mL of blood evacuated immediately following insertion, consistent with active intrathoracic hemorrhage. Due to retained hemothorax, a second chest tube was placed. The patient received four units of packed red blood cells during the operative course and required transient vasopressor support with phenylephrine for intraoperative hypotension, which was discontinued post-procedure.

*CPC-EM Capsule*

What do we already know about this clinical entity?

*Blunt thoracic aortic injury is a highly lethal trauma often associated with rapid deceleration and hemodynamic instability.*

What makes this presentation of disease reportable?

*This patient presented hemodynamically stable despite a grade IV aortic transection, highlighting that this severe injury may lack classic signs.*

What is the major learning point?

*High-risk mechanisms of injury should prompt early computed tomography imaging even when vital signs are normal.*

How might this improve emergency medicine practice?

*Maintaining suspicion for blunt thoracic aortic injury in stable trauma patients may lead to earlier imaging and improved survival.*

Postoperative laboratory testing demonstrated improving leukocytosis (20.2×10^9^/L) and an increase in hemoglobin to 13.1 g/dL following operative intervention and transfusion. On hospital day two, chest tubes were transitioned from suction to water seal following decreased output and stable chest radiographs. A computed tomographic angiography of the chest performed on hospital day 3 confirmed successful thoracic endovascular aortic repair placement with no endoleak and decreasing hemothorax. A small, stable left-sided pneumothorax was also noted. Due to retained hemothorax, intrapleural fibrinolytic therapy with tissue plasminogen activator and dornase alfa was later administered, after which chest tube output further declined; one chest tube was removed on hospital day 5 and the second on hospital day 6.

Subsequent review revealed that another occupant of the vehicle sustained a traumatic cardiac arrest and was diverted to a separate facility, information that was not initially available to the EMS team transporting this patient. This detail further underscores the severity of the collision and reinforces the importance of mechanism-based evaluation, even in patients who present with initially reassuring vital signs.

## DISCUSSION

Blunt aortic injury is typically associated with rapid deceleration injuries, and motor vehicle collisions are a common mechanism.^4^,^5^ Patients may present with normal hemodynamics.^6^ Therefore, eliciting detailed information about the mechanism of injury is essential. The most common site of injury is the aortic isthmus, followed by the aortic arch. 7 The grading scale ranges from grade 1 (intimal tear), grade 2 (intramural hematoma), grade 3 (aortic pseudoaneurysm), to grade 4 (free rupture).^8^

In this case, the unrestrained passenger status likely contributed to increased deceleration forces, producing the aortic transection. Notably, the patient arrived normotensive in the ED. This underscores the need for a high index of suspicion for blunt thoracic aortic injury, even in initially stable patients.

The diagnosis of blunt thoracic aortic injury in hemodynamically stable patients remains challenging, as initial signs may be nonspecific.^9^ Physical examination may show chest wall tenderness, abnormal breath sounds, or unequal pulses. An anteroposterior chest radiograph may reveal a widened mediastinum, tracheal deviation, rib fractures, or abnormal aortic contour. 10 However, these are not specific for blunt thoracic aortic injury, necessitating further imaging. 11 Computed tomography evaluation is considered necessary in high-risk motor vehicle collisions including unrestrained passengers at a speed of 10 mph, or restrained passengers at a speed of 30 mph.^11^ The Eastern Association for the Surgery of Trauma recommends considering blunt thoracic aortic injury in motor vehicle collision patients and using a chest radiograph as an initial screen while noting that significant deceleration mechanisms warrant further evaluation even with a normal mediastinum.^12^ In this case, the initial diagnosis was made using a contrast-enhanced CT of the chest for evaluation of suspected thoracic injury, which was sufficient to identify a large, grade 4, aortic transection and prompt immediate surgical consultation. However, CT angiography (CTA) remains the gold standard for diagnosis.^13^

Management of blunt thoracic aortic injury requires balancing injury severity with competing traumatic priorities. In hemodynamically stable patients where repair is delayed, medical management with anti-impulse therapy has been shown to safely reduce aortic wall shear stress. Fabian et al demonstrated that beta-blockade with or without vasodilators, targeting a systolic blood pressure of approximately 100 mm Hg and heart rate < 100 beats per minute, was associated with no observed aortic rupture while awaiting repair.^4^ These findings have been incorporated into Western Trauma Association guidelines, which recommend early anti-impulse therapy when definitive repair is delayed.^14^

The patient underwent successful thoracic endovascular aortic repair with no complications or endoleak. Contemporary guidelines from the Society for Vascular Surgery recommend endovascular repair over open or nonoperative management when anatomically feasible and suggest urgent repair, ideally within 24 hours after stabilization of other serious injuries.^15^ These guidelines incorporate established grading systems for blunt thoracic aortic injury, which classifies injuries from intimal tear to free rupture, and recommend nonoperative management with serial imaging for minimal (grade 1) injuries while favoring urgent endovascular repair for higher-grade (grade 2–4) injuries.^15^,^8^ Follow-up imaging with CTA confirmed repair integrity and decreasing hemothorax. Injuries near the aortic arch may require coverage of the left subclavian artery to achieve an adequate proximal seal. Current guidelines suggest selective rather than routine revascularization, individualized to patient anatomy and clinical context.^15^

This case underscores the importance of early, mechanism-driven imaging and a multidisciplinary approach in managing complex vascular trauma, particularly when the patient is stable on presentation. The involvement of trauma surgery, vascular surgery, interventional radiology, and critical care teams was integral to ensuring timely diagnosis and optimal outcomes.

## CONCLUSION

Blunt thoracic aortic injury remains a highly lethal injury requiring early recognition and rapid intervention. While commonly associated with hemodynamic instability, normotensive presentations demand a high degree of clinical suspicion. High-risk mechanisms of injury, such as an unrestrained motor vehicle collision, warrant significant concern for blunt thoracic aortic injury regardless of reassuring vitals. Imaging plays a pivotal role in diagnosis, with CTA serving as the gold standard. Early imaging and prompt surgical intervention, facilitated by a multidisciplinary team, are essential in improving survival outcomes.
